# Smoking and Religion: Untangling Associations Using English Survey Data

**DOI:** 10.1007/s10943-017-0434-9

**Published:** 2017-06-30

**Authors:** Manzoor Hussain, Charlie Walker, Graham Moon

**Affiliations:** 1 Social Sciences, University of Southampton, Southampton, UK; 2 Geography and Environment, University of Southampton, Highfield, Southampton, UK

**Keywords:** Religion, Smoking, Tobacco, Secondary analysis

## Abstract

While factors affecting smoking are well documented, the role of religion has received little attention. This national study aims to assess the extent to which religious affiliation is associated with current-smoking and ever-smoking, controlling for age, sex, ethnicity and socio-economic status. Variations between adult and youth populations are examined using secondary analysis of individual-level data from 5 years of the Health Survey for England for adult (aged >20, *n* = 39,837) and youth (aged 16–20, *n* = 2355) samples. Crude prevalence statistics are contrasted with binary logistic models for current-smoking and ever-smoking in the adult and youth samples. Analyses suggest that Muslims smoke substantially less than Christians. Highest levels of smoking characterise people not professing any religion. Associations between smoking and the Muslim religion attenuate to statistical insignificance in the face of ethnic and socio-economic factors. An association between smoking and the absence of a religious affiliation is sustained. An understanding of the association between smoking and religion is essential to the development of tobacco control programmes.

## Introduction

This paper explores the association of religion with smoking. In recent years, there has been increased interest in this topic (Chitwood et al. 2008; Anthony et al. 2013; Ford and Hill 2012; Garrusi and Nakhaee 2012; Karlsen et al. 2012; Karlsen and Nazroo 2010). Patterns of smoking are known to vary significantly by religion but less is known about how this association is affected by other factors or how, if at all, it differs between younger and older people. We address this gap in knowledge through a focussed case study of England, where recent falls in smoking prevalence have taken place alongside significant changes in religious affiliation but where the association between religion and smoking has received little attention. Understanding more about this association is potentially significant for the design of effective tobacco control interventions that take account of the specific needs and characteristics of religious groups while also reflecting the distinctiveness of populations of different ages.

Our motivation for focussing on the association between religion and smoking in England is twofold. First, as noted, recent years have seen the size of different religious groups in the England change markedly (ONS 2012). Between 2001 and 2011, the number of people identifying as Christian decreased by 13% (from 72 to 59%), while those who reported having no religion increased by 10% (from 15% in 2001 to 25% in 2011). Among the other main religions, the population of Muslims increased the most, from 3% in 2001 to 4.8% in 2011. To a significant extent, these changes reflect underlying demographics, hence our interest in comparing adult and youth populations in terms of smoking prevalence.

A second motivation relates to more theoretical and theological concerns. The major world religions have positions that are largely opposed to smoking (Khayat 2000; Garrusi and Nakhaee 2012). For example, within Christianity, Biblical interpretations condemn smoking as bodily pollution and an unnatural vice that runs counter to Christian values of temperance and moderation. Equally, Muslim perspectives are marked by leading clerics urging abstinence and pronouncing a *fatwa* against tobacco on the grounds of its potential to cause ill-health and offend Koranic injunctions to ensure personal health and the health of others. We ask whether such positions are evidenced in differential smoking prevalences between religious groups in the predominantly secular context of contemporary England.

Past studies of religion and smoking have generally focused on measures of religiosity, that is the depth or extent of religious belief. This body of work has very clearly pointed to higher levels of smoking among people who do not profess any religion and conversely lower smoking prevalences among religious people. Such associations have been found across much of the world, implicating Christian denominations, different forms of Islam and Eastern faiths. Research in the USA, for example, has linked greater religiosity with lower levels of smoking among both adults (Whooley et al. 2002; Garcia et al. 2013; Hayward et al. 2016; Bowie et al. 2017) and younger people (Alexander et al. 2016; Nonnemaker et al. 2006; Amey et al. 1996; Wallace and Forman 1998). Elsewhere similar conclusions have been drawn for young people in Central America and the Dominican Republic (Chen et al. 2004), Hungary (Kovacs et al. 2011), Switzerland (Becker et al. 2015), Iran (Ameri et al. 2016) and Jordan (Alzyoud et al. 2015), for adults in Brazil (Martinez et al. 2017), mainland China (Wang et al. 2015; Wang and Jang 2016), Zambia and Malawi (Pampel 2005) and South Africa (Prinsloo et al. 2008), and for pregnant women in San Luis, Brazil (Barbosa et al. 2015). Analogously, adults in South Korea have been found to be more likely to quit smoking if they are religious (Myung et al. 2012).

In terms of identification or affiliation with particular religions, a US study has suggested that tobacco use among Muslim college students is lower than that for non-Muslims (Ahmed et al. 2014). This finding is sustained for adult populations in the former Soviet Union (Pomerleau et al. 2004 and for pregnant Muslim women in Thailand who smoke less than pregnant Buddhist women (Assanangkornchai et al. 2017). Wang et al. (2015) link lower levels of smoking in China to more religiously observant Muslims. Ghouri et al. (2006), in contrast, link the Muslim religion to high and rising rates of smoking through a focus on national levels of smoking in ‘predominantly Muslim’ countries. Lakew and Haile (2015) find that Muslims (and Catholics) in Ethiopia smoke more than the people from the dominant Coptic Orthodox community. Chen (2014), in a Taiwanese study focussed on Eastern Religions, has made the important point that links between religious affiliation and smoking may not be robust to confounding.

Evidence focussed on the association between smoking and religion in England is sparse. It has drawn substantially on localised survey research in the West of Scotland highlighting the interplay of ethnicity, religion and life stage (Williams et al. 1994; Williams and Shams 1998; Bradby and Williams 2006). Youthful abstinent behaviour erodes earlier among non-Muslims and a higher prevalence of ever-smoking is evident among young Christians and ‘Others’ and a lower prevalence among young Muslims. This assessment broadly tallies with the conclusions of Anthony et al. (2013), who also used local survey data, showing lower ever-smoking and current-smoking prevalences in Leicester, England, among Muslims as compared to Christians and (more so) those who reported no religion. These differences in prevalence may reflect underlying beliefs: Francis (2008) suggests that, in England, 34% of young people not professing a religion believe that it is wrong to smoke compared to 39% of Christians and 54% of Muslims.

In the light of this current literature, we identify the need for national scale research that considers the impact of religion on smoking behaviour, contrasting youth and adult populations and controlling for potential confounding variables, particularly ethnicity.

## Methods

We used a secondary analysis approach with a cross-sectional research design contrasting data on youth and adult smoking behaviour drawn from the same source over a common time period.

### Data

We reviewed a number of candidate surveys but only the Health Survey for England (HSfE) covered all the variables needed to address our research questions simultaneously for both adults and young people. Some surveys covered only adults, and some only young people; others did not cover religious affiliation. Individual data from the Health Survey for England 2010–2014 (NatCen Social Research et al. 2013, 2014, 2015a, b, 2016) were downloaded from the UK Data Service. The HSfE is a cross-sectional survey carried out since 1991 and sponsored by the Health and Social Care Information Centre (now NHS Digital). The survey selects participants using a random probability sample and collects information through face-to-face interviews. It provides data on ethnicity, religion and smoking for both adults and young people. In order to enhance our sample size, we combined data from successive runs of survey from 2010 to 2014. To compare variations in the effect of religion on smoking for adults and youth, we worked with adult (aged >20, *n* = 39837) and youth (aged 16–20, *n* = 2355) samples.

### Measures

#### Smoking

We used two measures of smoking: ever and current. The ever smoked question asked respondents if they had ever smoked a cigarette, a cigar or a pipe. Respondents indicating ‘yes’ were classified as ever smokers, and those stating ‘no’ were classified as never smokers. The question captures people who have quit smoking have experimented with smoking and current smokers. In the current-smoking question, respondents were asked, do you smoke cigarettes at all nowadays? People answering yes were classified as current smokers, and those answering no were classified as non-smokers. This question isolates individuals currently classing themselves as a smoker. Neither question enables any conclusions to be drawn about the frequency of smoking. We did, however, construct an additional variable capturing respondents who had ceased to smoke, defined as ever smokers who were not current smokers.

#### Religion

Religion was recorded as a four category variable identifying respondents as Christian, Muslim, no religion or Other. The ‘other’ category amalgamated data on several religions for which numbers were too small to permit analysis. The heterogeneity within the ‘other’ category means that the analytical focus of the paper is on variations between Christians, Muslims and those professing no religion. We will not comment further on the ‘other’ category.

#### Confounder and Modifier Variables

We measured ethnicity by recoding the standard ethnicity variable from the HSfE into a single five-category variable. This was necessary as ethnicity was collected for several groups that were too small for the analysis. The recoded categories were White, Mixed, South Asian, Black and Other. We also included data on age, sex and socio-economic status. We measured age in years and used it as a continuous variable. Socio-economic associations were captured using data on whether or not an individual was in employment, and whether or not they possessed an educational qualification acquired after leaving school.

### Analyses

Our analyses used SPSS version 22. Descriptive statistics were used to calculate smoking prevalence and quit prevalence by religious group. Binary logistic regression was then performed to examine the associations between our dependent variable (ever/current smoking/quitting) and the exposure variable, religion, with controls for ethnicity, sex, age and socio-economic status. Analyses were conducted separately for the youth and adult samples. We set the contrast category for religion to be ‘none’, enabling us to explore the extent to which religion is associated with higher or lower probabilities of smoking or quitting.

In order to know if the confounding or modifying variables affect the association between smoking and religion, we built our model sequentially beginning with an age, sex, religion model, then adding ethnicity, and finally incorporating the socio-economic variables. We tested for multicollinearity using tolerance levels and the variance inflation factor (VIF) and found no issues. We also assessed two- and three-way interactions between religion, ethnicity and our socio-economic variables in all models, and none were significant. In view of small sample size in the youth study, bootstrapped standard errors were used to adjust odds ratios. Our analysis of quitting considered only the adult sample as smoking cessation among youth is a fluid process reflecting experimentation with tobacco as well as genuine cessation, and sample sizes were too small for meaningful analysis.

## Results

### Descriptive Statistics

Descriptive statistics suggest clear differences in ever-smoking and current-smoking prevalence, and quit prevalence by religion (Table 1). There were higher ever-smoking prevalences among adults who reported no religion (66.2%) and Christians (60%), and relatively lower prevalences among Muslim (35.2%) adults. Current-smoking prevalence by religion shows a different pattern, with almost similar levels among Christian (16.7%) and Muslim (16.9%) adults, and higher levels among adults who do not belong to any religion (25.1%). Muslim adults were the least likely to quit smoking (50.59%), by a substantial margin, while Christians were most likely to quit (73.9%). In the youth sample the highest ever-smoking and current-smoking prevalences are both recorded among youth reporting no religion (53 and 25.3%, respectively). Christian youth have prevalences approximately 10% lower (42.2 and 16.7%, respectively), while lowest levels are among Muslim youth (18.6 and 5.8%). Christian youth and youth with no religion have similar levels of current-smoking to their adult counterparts. In contrast, Muslim youth return a current-smoking prevalence less than one-third that of Muslim adults.

**Table 1:** Smoking prevalence by religion; HSfE, 2010–2014

Religion	Adult						Youth			
	Ever smoked		Current smoker		Quitter		Ever smoked		Current smoker	
	%	*n*	%	*n*	%	*n*	%	*n*	%	*n*
Christian	60.0	20,974	16.7	23,516	73.9	12,584	42.2	688	16.7	828
Muslim	35.2	959	16.9	1153	50.6	338	18.6	102	5.8	137
None	66.2	8720	25.1	10,027	65.1	5776	53.0	886	25.3	1048
Other	41.7	1229	12.6	1439	69.7	512	25.6	82	7.6	105

*n* = total sample in each category, i.e. ever plus never; current plus not current; quitter plus non-quitter

### Modelling the Association Between Smoking and Religion

#### Current-Smoking

Table 2 examines current-smoking, comparing adult and youth samples. Model 1 offers broad confirmation of the initial descriptive finding discussed above. Controlling for age and sex, Muslim respondents are significantly less likely than Christians to be current smokers in both the adult and youth samples. Odds ratios less than one indicate that all religions are associated with probabilities of current-smoking below that for adults or youth who do not profess a religion. Models 2 and 3 trace how these associations change with the addition of ethnicity and socio-economic factors as modifier and confounder variables. In the adult sample, the suggestion that Muslims smoke more than people with no religion attenuates to statistical insignificance once ethnicity and socio-economic factors are controlled. The evidence in the youth models is less straightforward: although the apparent association between being Muslim and lower current-smoking attenuates with both ethnicity and socio-economic status, it disappears only when socio-economic factors are taken into account. In contrast, for Christians, the association with a lower probability of current-smoking is maintained for both adults and youth in the face of confounders and modifiers.

**Table 2:** Modelling smoking and religion, current-smoking, HSfE 2010–2014: odds ratios (95% confidence intervals); italicised denotes non-significance

	Adult			Youth		
	Model 1	Model 2	Model 3	Model 1	Model 2	Model 3
*Religion*						
None						
Muslim	0.54 (0.47–0.64)	*1.02 (0.83*–*1.26)*	*0.76 (0.52*–*1.11)*	0.18 (0.07–0.33)	0.37 (0.13–0.77)	*0.51 (0.11*–*1.54)*
Christian	0.80 (0.75–0.85)	0.82 (0.77–0.87)	0.86 (0.77–0.95)	0.60 (0.46–0.76)	0.62 (0.47–0.79)	0.64 (0.41–0.94)
Other	0.45 (0.39–0.54)	0.70 (.0.58–0.84)	0.72 (0.52–0.99)	0.24 (0.09–0.42)	0.45 (0.16–0.89)	*0.41 (0.01*–*1.23)*
*Sex*						
Man						
Woman	0.83 (0.78–0.87)	0.83 (0.78–0.87)	0.79 (0.72–0.87)	*0.90 (0.72*–*1.14)*	*0.90 (0.72*–*1.14)*	*0.88 (0.62*–*1.30)*
*Age*						
Years	0.98 (0.97–0.98)	0.97 (0.97–0.98)	0.96 (0.96–0.96)	1.30 (1.20–1.40)	1.30 (1.21–1.41)	1.45 (1.26–1.68)
*Ethnicity*						
White						
Mixed		*1.08 (0.94*–*1.23)*	*0.77 (0.60*–*1.00)*		*1.48 (0.90*–*2.32)*	*1.21 (0.45*–*2.67)*
South Asian		0.43 (0.35–0.53)	0.40 (0.28–0.58)		0.19 (0.04–0.51)	0.09 (0.00–0.46)
Black		0.46 (0.37–0.56)	0.44 (0.31–0.61)		0.21 (0.04–0.49)	0.08 (0.00–0.31)
Other		0.57 (0.45–0.73)	0.46 (0.31–0.69)		*1.05 (0.41*–*2.34)*	*0.88 (0.00*–*3.06)*
*Employed*						
Yes						
No			0.92 (0.79–1.06)			0.56 (0.36–0.83)
*Post-16 Qualifications*						
Yes						
No			2.47 (2.20–2.77)			*1.70 (0.81*–*3.22)*

The other variables in Table 2 conform to expectations. Women are less likely to be current smokers in both the adult and youth samples, though in the youth sample the association is not significant. The odds of being a current smoker reduce with age for adults but increase in the youth sample; older people are giving up as they age, while younger people are moving from experimentation to smoker status. People, both adults and youth, of mixed ethnicity are indistinguishable from those of White ethnicity in terms of their odds of being a current smoker. South Asians and Blacks are significantly less likely to be current smokers compared to Whites, and the association is far stronger in the youth sample. Young people who are unemployed (and consequently with low financial resources) are significantly less likely to be current smokers, while adults without educational qualifications are significantly more likely to be current smokers. Comparisons between the odds ratios for religion, ethnicity and socio-economic facts suggest that associations between religion and current-smoking are possibly very slightly more impacted by socio-economic factors in the adult sample and by ethnicity in the youth sample.

#### Ever-Smoking

In Table 3, we present the results for our analysis of ever-smoking. This variable provides a more expansive definition of smoking. In comparison with current-smoking, it captures, in broad terms, the extent of quitting among adult smokers and experimentation in our youth sample. There are, however, many similarities between the results for the two measures of smoking behaviour. Model 1 suggests, for both adults and youth, that Muslims are less likely than Christians to have ever smoked; people without a religion are more likely to have smoked. The significant association between the Muslim religion and ever-smoking only attenuates to non-significance with the inclusion socio-economic confounders and moderators for both adults and youth. The significant association with Christianity attenuates completely in the youth sample removing any suggestion that Christianity has an association with never having smoked. The suggestion that Christians are less likely to smoke than people with no religion persists in the adult sample. A comparison of the patterns of attenuation associated with religion with those associated with ethnicity and socio-economic factors suggests that ethnicity may be more instrumental than socio-economic factors in the attenuation of the Muslim effect for adults, while ethnicity and socio-economic factors are equally relevant in the attenuation for youth.

**Table 3:** Modelling smoking and religion, ever-smoking, HSfE 2010–14: odds ratios (95% confidence intervals); bootstrapped for youth sample; italicised denotes non-significance

	Adult			Youth		
	Model 1	Model 2	Model 3	Model 1	Model 2	Model 3
*Religion*						
None						
Muslim	0.29 (0.25–0.33)	0.68 (0.56–0.82)	*0.74 (0.54*–*1.02)*	0.20 (0.11–0.32)	0.45 (0.22–0.89)	*0.76 (0.15*–*3.83)*
Christian	0.74 (0.70–0.79)	0.77 (0.73–0.81)	0.82 (0.75–0.91)	0.65 (0.52–0.79)	0.67 (0.54–0.83)	*0.80 (0.59*–*1.17)*
Other	0.37 (0.32–0.41)	0.67 (0.58–0.77)	*0.85 (0.66*–*1.10)*	0.30 (0.16–0.48)	*0.61 (0.32*–*1.06)*	*1.22 (0.41*–*3.81)*
*Sex*						
Man						
Woman	0.61 (0.59–0.64)	0.61 (0.58–0.64)	0.62 (0.57–0.68)	*0.94 (0.77*–*1.13)*	*0.94 (0.77*–*1.13)*	*0.84 (0.59*–*1.22)*
*Age*						
Years	1.01 (1.01–1.01)	1.01 (1.00–1.01)	1.01 (1.00–1.01)	1.14 (1.07–1.22)	1.15 (1.07–1.23)	1.16 (1.02–1.32)
*Ethnicity*						
White						
Mixed		*1.08 (0.96*–*1.22)*	*1.07 (0.86*–*1.34)*		*1.22 (0.80*–*1.85)*	*1.12 (0.45*–*2.90)*
South Asian		0.32 (0.27–0.38)	0.29 (0.22–0.39)		0.25 (0.12–0.48)	0.14 (0.02–0.47)
Black		0.31 (0.26–0.36)	0.28 (0.21–0.37)		0.34 (0.16–0.59)	0.11 (0.00–0.28)
Other		0.48 (0.40–0.58)	0.40 (0.29–0.54)		*0.79 (0.32*–*1.70)*	*0.21 (0.00*–*1.04)*
*Employed*						
Yes						
No			*0.90 (0.80*–*1.02)*			2.14 (1.40–3.40)
*Post-16 Qualifications*						
Yes						
No			1.60 (1.44–1.78)			*1.09 (0.62*–*2.04)*

Similarities with the current-smoking models also extend to the results for the confounder and moderator variables. Adult women are less likely than men to have ever smoked, South Asian and Black ethnicity is associated with lower levels of smoking than the White reference group for both adults and youth, and adults without qualifications are more likely to have ever smoked. Differences are evident in the results for age where both the adult and youth sample are more likely to be ever smokers with increasing age, reflecting experimentation in the youth sample and age-related quitting among the adults. The association of unemployment with ever-smoking in the youth sample also differs: while unemployed youth were less likely to be current smokers, they are significantly more likely to have ever smoked.

Modelling of adult quitting behaviour revealed only that the likelihood of quitting increased with age (OR 1.05, 95% CI 1.04–1.06) and was lower for people lacking a post-16 educational qualification (OR 0.49, 95% CI 0.43–0.57). In the full model 3 formulation, the likelihood of quitting did not differ significantly between Muslims, Christians and those not professing a religion. Indeed, there was no indication that the effect for Christians was different from that for those with no religion in any model. A suggestion in the model 1 and 2 formulations that Muslims are statistically less likely to quit than people with no religion proved in model 3 to be an artefact of their socio-economic status.

## Discussion

In contrast to previous UK studies that have focussed on specific locations (the West of Scotland or Leicester), the present study has provided national evidence for England comparing three measures of smoking behaviour between youths and adults and highlighting the extent of association with religion while controlling for other relevant factors. Our findings respond to concerns about confounding articulated by Chen (2014) in a very different national context and develop and enhance suggestions by Bradby and Williams (2006) and Anthony et al. (2013) about the interplay of ethnicity, religion and socio-economic status in understanding smoking behaviour.

Initial indications from simple cross-tabulations suggested that Muslim youth are far less likely to be current smokers than their Christian or no-religion counterparts. This confirms evidence from the US, China and the former Soviet Union (Ahmed et al. 2014; Pomerleau et al. 2004; Wang et al. 2015). Moreover, Muslim youth are less likely to be current smokers in comparison with Muslim adults; this discrepancy is not evident for Christians, sustaining Frances’ (2008) argument that Muslim youth are particularly likely to deem smoking to be wrong. Muslim adults and youth also stand out as being less likely to have ever smoked. These simple associations suggest that the research in the West of Scotland pointing to abstinence persisting longer among Muslim youth (Bradby and Williams 2006) may have wider relevance to England.

Across both adult and youth groups, simple descriptive analyses pointed to smoking (both ever and current) being highest among people professing no religion. This confirms that the widely held global equation of lower religiosity with higher levels of smoking applies to England and adds to knowledge by demonstrating that this finding is relevant beyond adolescent English populations (Francis 2008) and the City of Leicester (Anthony et al. 2013). This position is sustained our simplest models, indicating that it is not an artefact of age or sex. Both Christians and Muslims appear to be less likely to smoke than people with no religion with Muslims generally being particularly averse. This initial finding gives strength to suggestions that religion may somehow protect against smoking, perhaps by binding its adherents in social communities with shared norms of abstinence and obedience to recommendations by leaders, as well as scope for mutual support (Gryczynski and Ward 2011; Mason et al. 2012). Wray-Lake et al. (2012) in national repeated cross-sectional study of US adolescents has shown how such social capital constructs have independent negative associations with smoking.

Our analysis of quitting challenges this conclusion. If religion points towards a lower smoking prevalence, we would expect that it might also point to higher levels of smoking cessation. While this is the case with Christianity, it is not evident with the Muslim religion. In a simple cross-tabulation, Muslim adults are less likely to quit smoking than adults declaring that they do not identify with any religion. It is well established that smoking cessation and continued smoking are distinct processes (Hyland et al. 2006) so it would be entirely possible for religion to simultaneously assist individuals in stopping smoking initiation while also hindering quitting. Why it might work differentially for Christians and Muslims is unclear. Croucher and Choudhury (2007) offer potential insights with their suggestion, based on qualitative work, that continued smoking among Muslims reflects anxieties about harassment, low-status employment, and the long shadow of migration experiences. Though these factors are undoubtedly significant for Muslims, they are not, however, exclusive to Muslims. Potentially more pertinent is the possibility that smoking provides a counter to the stresses and strains of being a minority religion. Padela and Curlin (2013) have developed this argument in the US context in relation to a range of health conditions and it draws strength from established theories about relative inequality and health behaviour (Jen et al. 2009). To unpack these possibilities, we need to turn to our modelling analyses.

Our models add to knowledge by demonstrating that, in England, our initial finding of an association between smoking and the presence of a religious affiliation is generally robust to confounders and moderators only in the case of Christianity. This conclusion suggests that the hypotheses linking religious social capital to smoking cited above may be relevant in England within a Christian context. The association with Christianity applies to current-smoking by both adults and youth and to adult ever-smoking. With our youth sample we were, however, unable to demonstrate a statistically significant association between ever-smoking and a Christian affiliation. In contrast, the initial associations linking the Muslim religion to low levels of smoking and also paradoxically to low levels of quitting are not robust to the impact of other relevant variables. We are thus unable to sustain the relative inequality/minority religion hypothesis. Ethnicity and, particularly, socio-economic factors trump the effect of religious affiliation on smoking prevalence for Muslims in England. Socio-economic status also over-rides any suggestion that Muslims are less likely to quit smoking. It is also clear that religious social capital is, at least in England, not a significant factor in smoking cessation, either for Muslims or Christians. This conclusion echoes that found in the very different context of Thailand by Yong et al. (Yong et al. 2009, 2013) who have emphasised that religion and religious authority are both potentially important in driving smoking cessation but neither ensure success, particularly in secular societies.

Our study has strengths and limitations. We present evidence from linked runs of a well-found long-established routine national survey using appropriate statistical methods and standard measures of smoking behaviour. However, despite merging 5 years of data, our sample size remained relatively small and led us to employ broad and potentially confusing ethnic categorisations. ‘South Asian’ and ‘Black’, for example, cover very diverse communities and there is no clear congruency between our ethnic and religious categorisations. Equally, we were unable to separate out different forms of Christianity or Islam. Small numbers are also evident in our youth samples though potential shortcomings have been addressed through a bootstrapped analysis. A further limitation is, of course, the cross-sectional design of our study. As a consequence, we do not seek to draw conclusions regarding the causal nature of the association between religion and smoking.

The potential implications of our study concern both future research and the practice of tobacco control. An enhanced qualitative component to future research will be essential if we are to explore more fully the relationship between religion and smoking. In-depth information drawing on interviews, ethnographic observation and the voices of different religious groups (and the non-religious) will be needed to draw out the extent to which people understand the impact of religion on smoking initiation, cessation and maintenance, and its interaction with other factors. Equally quantitative longitudinal studies are also needed to trace the interplay of religion, smoking and other confounding and moderating factors over time.

In terms of tobacco control, our results raise issues for faith-based health interventions. Evidence primarily from the USA but also from the Far East and Muslim countries has been hopeful but equivocal about the effectiveness of such measures (Campbell et al. 2007; Schoenberg et al. 2016; Ismail et al. 2016; Byron et al. 2015; Elkalmi et al. 2016). Our research points to the need for faith-based interventions to move beyond baseline prevalences to understand how religion interacts with other factors that may be more important in driving smoking behaviour, notably socio-economic disadvantage and ethnicity. We also underline the importance of targeting those without a religious faith and recognising that the association between smoking and religion is not uniform across all faiths. The potential for effective faith-based interventions in England would appear to be greatest for interventions based around Christian congregations drawing strength from the independent association of Christianity with lower smoking prevalences. There is, however, potential for all faiths provided it is recognised that religion is both more complex in terms of its role as an epidemiological construct (Levin 1996) and more complex than is commonly understood in health promotion (Liu et al. 2016). As Ward et al. (2014) note the link between religion and smoking can vary significantly across different religious communities and must (Schoenberg et al. 2015) be deployed with careful attention to community norms if it is to be effective.
